# The development and validation of an artificial intelligence-based screening method for atrial septal defect in children's chest x-rays

**DOI:** 10.3389/fped.2023.1203933

**Published:** 2023-09-11

**Authors:** Li Zhixin, Luo Gang, Ji Zhixian, Pan Silin

**Affiliations:** Heart Center, Women and Children’s Hospital, Qingdao University, Qingdao, China

**Keywords:** artificial intelligence, screening method, chest x-ray, congenital heart disease, atrial septal defect

## Abstract

**Purpose:**

For precise diagnosis and effective management of atrial septal defects, it is of utmost significance to conduct elementary screenings on children. The primary aim of this study is to develop and authenticate an objective methodology for detecting atrial septal defects by employing deep learning (DL) on chest x-ray (CXR) examinations.

**Methods:**

This retrospective study encompassed echocardiographs and corresponding Chest x-rays that were consistently gathered at Qingdao Women's and Children's Hospital from 2018 to 2022. Based on a collaborative diagnosis report by two cardiologists with over 10 years of experience in echocardiography, these radiographs were classified as positive or negative for atrial septal defect, and then divided into training and validation datasets. An artificial intelligence model was formulated by utilizing the training dataset and fine-tuned using the validation dataset. To evaluate the efficacy of the model, an assessment of the area under the curve, sensitivity, specificity, accuracy, positive predictive value, and negative predictive value was conducted employing the validation dataset.

**Results:**

This research encompassed a total of 420 images from individuals. The screening accuracy and recall rate of the model surpass 90%.

**Conclusions:**

One of profound neural network models predicated on chest x-ray radiographs (a traditional, extensively employed, and economically viable examination) proves highly advantageous in the assessment for atrial septal defect.

## Graphical Abstract

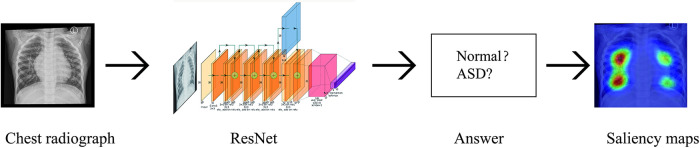
 Model schematic.

## Introduction

Atrial septal defect (ASD) is the most common type of congenital heart disease, including several types of interatrial communication defects that allow blood shunting between the systemic and pulmonary circulation (1). There are five types of atrial septal defects ranging from most frequent to least: patent foramen ovale, ostium secundum defect, ostium primum defect, sinus venosus defect, and coronary sinus defect (2, 3). Most children with isolated ASD do not display symptoms. However, as they age, they are at risk for decreased exercise tolerance, atrial arrhythmias, right ventricular dysfunction, and pulmonary hypertension. Untreated adult patients with a defect have a reduced life expectancy, and females or those of advanced age with untreated defects have an increased risk of developing pulmonary vascular disease, a potential lethal complication (4). Surgical intervention is a safe and effective method to address ASD (5). Surgery before the age of 25 results in a life expectancy equivalent to that of the general population (6). Therefore, early detection and treatment are crucial management principles (7).

Currently, the commonly employed cardiovascular diagnostic methods in clinical practice encompass electrocardiography, chest radiography, echocardiography, magnetic resonance imaging (MRI), and computed tomography (CT). Echocardiography has emerged as the gold standard for diagnosing atrial septal defects due to its non-invasive, convenient, and intuitive nature. However, interpreting the images of echocardiography requires experienced cardiac sonographers as there are significant variations among different imaging planes, making standardized interpretation difficult and rendering it inadequate as an ideal screening tool. Electrocardiography lacks specificity and may pose challenges in uncooperative children, while being sensitive to external chest factors such as the lungs, thoracic cavity, and chest wall. MRI and CT, on the other hand, are expensive and not ideal screening tools.

Chest radiography provides a simple, rapid, non-invasive, and cost-effective screening method for detecting atrial septal defects. In the chest radiography, certain characteristic findings can be observed, such as enlargement of the right atrium and pulmonary artery, especially in the anterior-posterior view, while right ventricular enlargement can be seen in the lateral view. Likewise, left atrial enlargement and distension (related to mitral regurgitation in type II congenital atrial septal defects) are also evident in the lateral view. The marked size differences between the pulmonary artery and relatively fewer peripheral blood vessels may indicate pulmonary vascular obstructive diseases (8, 9). These discoveries prompt further examination and confirmation by clinical practitioners. Therefore, we have chosen chest radiography as a more ideal screening method for children with atrial septal defects.

Machine learning and computer vision technologies provide the means to enhance insight, increase accuracy, and optimize workload times for interpretation purposes. With the improvement in both the quality and availability of medical imaging equipment, alongside the promotion of effective healthcare policies, medical imaging has become a critical step in modern medical diagnosis and treatment. The interpretation of medical imaging requires specialized training and can be time-consuming. Traditional machine learning techniques in medical imaging involve matching features designed by domain experts, which is a tedious and limited process. Recent advances in deep learning techniques, coupled with the increasing prevalence of powerful Graphics Processing Units (GPUs), allow for data-driven approaches that make image-based diagnosis automation possible (10, 11). One particular area of interest is the development of AI-based screening methods for the early detection of cardiovascular conditions in children. Among these conditions, atrial septal defect stands as a significant concern due to its prevalence and potential complications. The aim of this study was to develop and validate an AI-based screening method specifically tailored for the detection of atrial septal defect in children's chest x-rays. Through the utilization of the potent force of artificial intelligence, this research strives to heighten the detection rate of children afflicted with ASD. Consequently, it aids physicians in expeditiously and promptly identifying cases of ASD, ultimately contributing to the enhancement of patients' treatment outcomes.

## Materials and methods

### Study design

We have developed a deep learning-based model that uses digital chest x-ray to classify Atrial septal defect. We retrospectively collected chest x-ray images from pediatric patients diagnosed with ASD at our hospital. The diagnosis of Atrial Septal Defect in a patient is determined by two experienced sonographers with over a decade of cardiac ultrasound expertise. They assess the two-dimensional echocardiogram to confirm the presence of ASD. We then established a classification neural network model using deep learning techniques. Subsequently, we applied classification activation maps to identify the regions of interest in the chest radiographs. The protocol of our study was reviewed and approved by the ethics committee of our institution. Because these images were obtained from patients who consented to the comprehensive research use of their data during routine clinical practice, the need for informed consent was waived. Patients were assured the opportunity to opt out of the study.

### Data partition

The dataset used in this study was collected from Qingdao Women's and Children's Hospital, consisting of digital radiography results of 420 children divided into a normal group and an atrial septal defect group. To ensure data quality, we included patients in the dataset whose time interval between chest x-ray and echocardiographic examinations did not exceed 5 days. The x-ray images were saved in JPG format and underwent operations such as cropping and rotation during model training to ensure data quality and matched sample sizes through data preprocessing.

The dataset included clear radiography results of children with normal examinations or atrial septal defects before surgery. The exclusion criteria included congenital chest/lung abnormalities, pulmonary infections or lesions, right-sided heart, post-cardiac surgery, lateral or oblique images, and other factors that may affect image quality.

### Image acquisition

In this study, retrospective posteroanterior chest x-ray of patients were obtained using the DRX Evolution Plus (Carestream Health, USA) imaging system after diagnosis by a treating clinician. All radiographs that met the appropriate diagnostic criteria for ASD were collected for analysis. This approach ensured that all included radiographs were clinically relevant and met the necessary standards for accurate diagnosis.

### Data partitioning

To facilitate the training and evaluation of the deep learning models, the labelled chest x-ray were partitioned into distinct training and validation datasets in an 8:2 ratio. Through the partitioning of our dataset into distinct training, validation sets, the models were trained and evaluated on independent subsets of data, leading to a more precise and reliable evaluation of their diagnostic performance. This careful partitioning approach helped eliminate potential confounding variables that may have impacted the accuracy and robustness of our findings.

### Model development

We built a ResNet18 model using the PyTorch framework (12, 13). The model was trained using the transfer learning method with the training dataset and fine-tuned using the validation dataset to identify features that distinguish between ASD positive and ASD negative images during training. To enhance the images, we applied random rotation, random shift, and brightness shift (14–16). The best-performing model was selected as the one with the minimum loss function value in the validation dataset during 50 epochs.

### Visualizing regions of interest for the trained model by using heat maps

To enhance the classification performance of the best-performing deep learning model in detecting ASD in chest x-ray, a heat map was generated for each radiograph to visualize its areas of focus. This was achieved using a classification activation map that applied global average pooling on the last convolutional layer of the model (17). By utilizing the trained weights for each output from the global average pooling layer, the relevance and importance of each feature map from the last convolutional layer were determined. Subsequently, these weights were applied to the corresponding feature maps, which were then superimposed on the original chest x-ray. The resulting class-discriminative visualization allowed for a more comprehensive understanding of the model's decision-making process (18).

### Statistical analysis

In this study, the performance of various models was assessed using several key metrics, including sensitivity, specificity, accuracy, positive predictive value (PPV), negative predictive value (NPV), and the area under the receiver operating characteristic curve (AUC). To ensure the reliability of the metric estimates, a statistical approach based on the Clopper-Pearson method was employed to calculate 95% confidence intervals for the aforementioned performance metrics. This approach provides a measure of uncertainty around each estimate and allows for a more robust evaluation of model performance.

### Role of the funding source

The funding source for this study did not participate in the design of the study, collection, analysis, interpretation of data or preparation of the report. The corresponding author had unrestricted access to all data generated during the study and had the final responsibility to decide on the publication of the manuscript.

## Results

### Datasets

**Figure 1:**
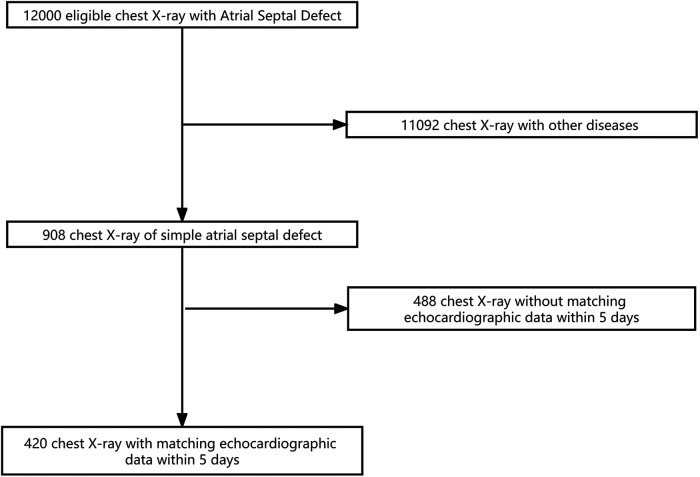
Review and allocation flowchart.

A total of 420 x-ray images and 420 corresponding echocardiographic examination reports from 420 patients were used in this study. The training dataset consisted of 336 images (336 patients; age range: 1–10 years, mean ± SD: 4.5 ± 1.5 years). The validation dataset consisted of 84 images (84 patients; age: 1–10 years, mean ± SD: 4.9 ± 1.9 years). The flowchart of the dataset standard process is shown in Figure 1. The dataset information is shown in Table 1.

**Table 1:** Dataset demographics.

	Training dataset	Validation dataset
Total no. of radiographs	336	84
Total no. of echocardiography	336	84
Total no. of patients	336	84
Male	155	45
Female	181	39
Mean age (years ± SD)	4.5 ± 1.5	4.5 ± 1.9
Mean period between examinations (days ± SD)	2 ± 1	2 ± 1

### Model development

The independent development of each model in this study involved training them for 50 epochs on the provided training dataset, followed by evaluation based on their loss value on a separate validation dataset. To ensure a fair comparison, identical hyperparameters were used across all models, including the Adagrad optimizer, an image size of 500 pixels, three channels, and global average pooling.

These specific hyperparameters were selected based on prior experimentation and were found to yield optimal performance. Consistent use of these parameters across all models helped eliminate confounding factors that could potentially impact the model's performance, enabling a more accurate assessment of their relative diagnostic capabilities. Overall, this approach ensured a rigorous and systematic evaluation of each model's performance and minimized any sources of bias or variability.

### Model evaluation

The radiographic images in this study were classified into two distinct categories, namely Normal, ASD, using an artificial intelligence-based classification model. The model's performance was evaluated by comparing its output with the ground truth labels assigned to each image. To assess the model's accuracy more comprehensively, performance metrics such as precision, recall, F1-score, etc., were calculated for each category (Table 2).

**Table 2:** The effect of the model.

Type	Precision	Recall	F1-score	Support	Accuracy	AP	AUC
ASD	0.971	0.895	0.932	38.000	0.895	0.982	0.981
Normal	0.909	0.976	0.941	41.000	0.976	0.982	0.981
Macro avg.	0.940	0.935	0.936	79.000	0.935	0.982	0.981
Weighted avg.	0.939	0.937	0.937	79.000	0.937	0.982	0.981

**Figure 2:**
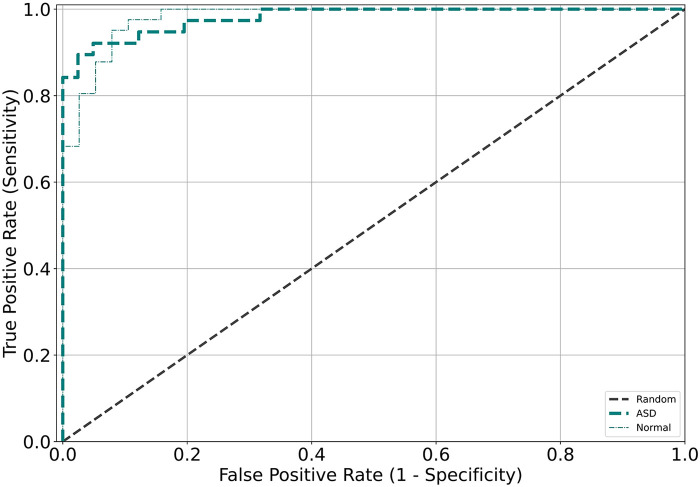
Receiver operating characteristic (ROC) curves were generated for the validation and test datasets for each model, with different colors corresponding to the different validation datasets.

**Figure 3:**
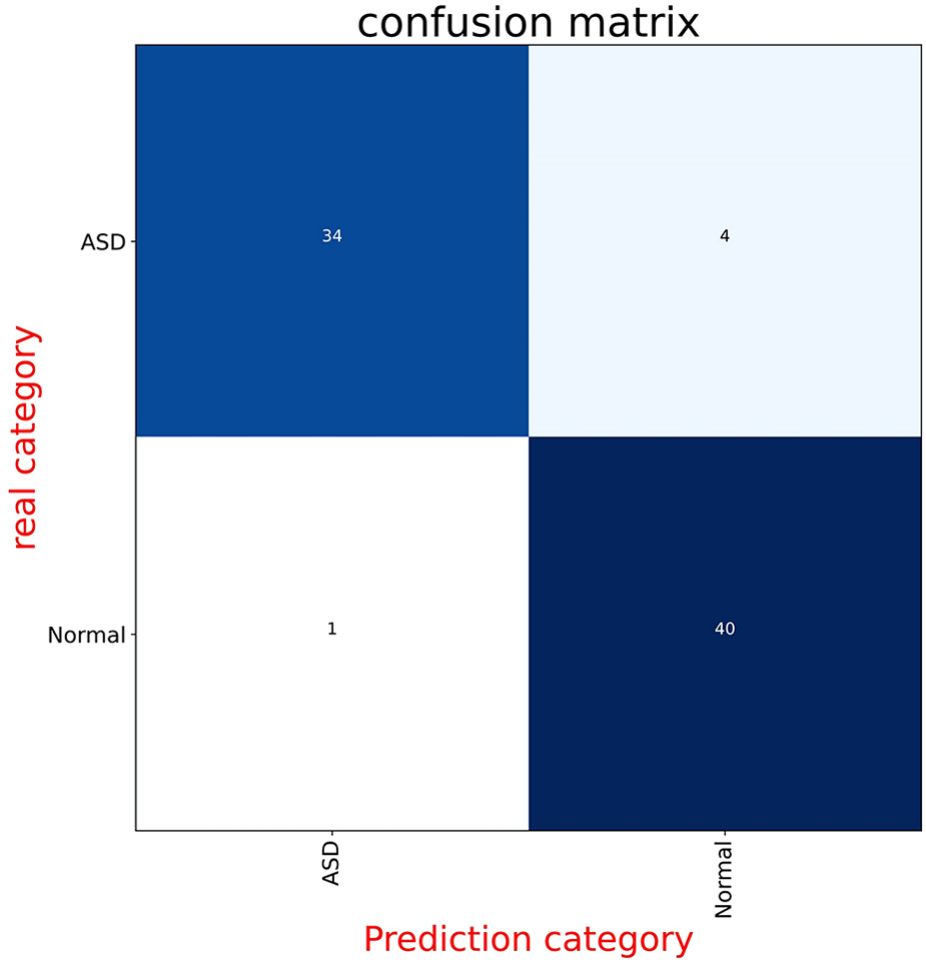
These panels display the confusion matrices for the model on both the validation and test datasets. Each figure is divided into four parts, with the numbers indicating the quantity of radiographs. The background color changes to a darker blue as the number of radiographs increases.

Figure 2 displays the receiver operating characteristic (ROC) curve, which is a graphical representation of the true positive rate versus the false positive rate of the model's predictions. This curve provides an intuitive measure of the model's ability to accurately discriminate between different categories. On the other hand, Figure 3 presents the confusion matrix, which is a table that summarizes the model's classification performance across all categories. The rows and columns of the matrix correspond to the predicted and actual labels, respectively.

**Figure 4:**
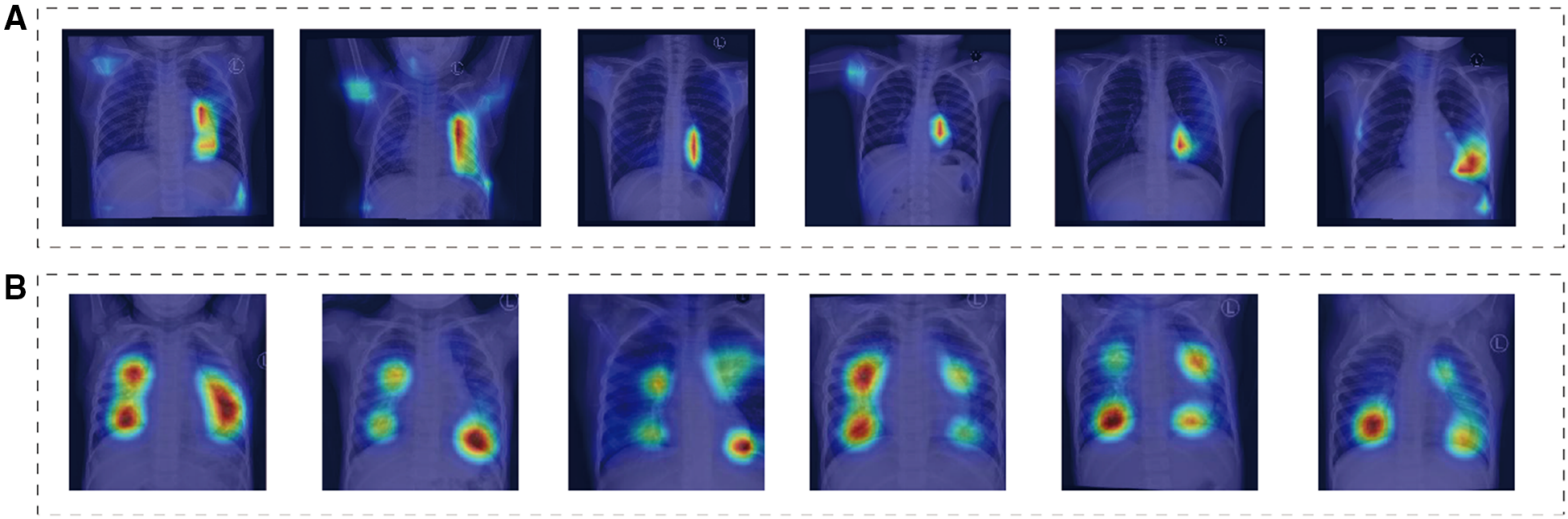
These saliency maps illustrate the features of chest x-ray that were important for the model to correctly diagnose normal, ASD. The heat maps highlight the areas of the radiographs that the model focused on when making the diagnosis. (**A,B**) Depict chest x-ray of children, showcasing normal radiographs, as well as radiographs of children with ASD.

Additionally, Figure 4 depicts the saliency maps of the top-performing models, which highlight regions of the input image that the model pays more attention to when making its prediction (19–21). These maps provide valuable insights into the model's decision-making process and can aid in identifying areas for improvement or potential biases.

## Discussion

Advanced deep learning techniques were utilized in this investigation to develop a screening model capable of detecting the likelihood of ASD from chest x-ray. The highest-performing model exhibited exceptional screening accuracy, achieving an AUC exceeding 0.93 on both the validation and test datasets, indicating high sensitivity and specificity. The results highlight the potential use of chest x-ray as a valuable tool for screening individuals for ASD, especially in resource-limited settings where other imaging modalities may not be readily available or feasible. Importantly, these findings provide evidence supporting the value of machine learning-based approaches in healthcare, as they can significantly improve screening accuracy and efficiency. Moreover, this study represents the first attempt to establish a screening model for ASD based on chest x-ray. By utilizing heat maps, specific regions on chest x-ray indicative of the likelihood of these conditions were identified, providing novel insights into the underlying pathophysiology of these diseases.

This knowledge could ultimately lead to improved patient outcomes through earlier detection and intervention. Expanding the chest radiograph dataset for cardiovascular disease has the potential to offer modeling advantages. Furthermore, our proposed model could prove to be a valuable asset given the challenges faced by pediatric patients who are unable to undergo traditional echocardiographic examinations. Notably, utilizing chest x-rays as an alternative can enable faster diagnosis and treatment.

It is important to recognize that additional research is required to validate the effectiveness and precision of this approach. The limitations of this study, including its retrospective design and the requirement for prospective multicenter investigations, highlight the necessity for ongoing efforts to improve and optimize this model. Nonetheless, our findings demonstrate encouraging progress in medical machine learning, showcasing the potential for novel strategies to advance clinical outcomes and diagnosis. With adequate validation and refinement, the proposed model has the potential to serve as a valuable tool for improving patient care, particularly for pediatric patients who face difficulties with traditional diagnostic methods.

The retrospective design of this study introduces inherent bias and may impact the generalizability and reliability of its findings. While heat maps were utilized as a means of visualizing regions of interest in the model, the precise features and criteria utilized by the algorithm to determine the clinical significance of these areas are currently unclear. Retrospective studies, such as this one, have limitations that must be taken into account when interpreting their results. These include potential confounding factors, selection bias, and recall bias, among others. As such, caution should be exercised when drawing conclusions from these types of investigations. Although heat maps can provide valuable insights into patterns of activity or association on imaging studies, their interpretation requires a thorough understanding of the underlying mechanisms that give rise to these patterns. Without this knowledge, the clinical relevance and utility of identified regions may be limited. In light of these considerations, it is important to further explore the diagnostic and prognostic potential of medical machine learning approaches while carefully considering the limitations of retrospective studies and the challenges associated with the interpretation of complex imaging data.

In conclusion, our study has demonstrated the successful development of a sophisticated deep learning-based artificial intelligence model with the ability to accurately diagnose ASD. The clinical implications of this model are promising, as it holds significant potential to assist physicians in making diagnostic decisions for patients with these conditions. Nonetheless, it is imperative to acknowledge that further research is needed to validate these findings. Prospective multicenter studies are particularly important to confirm the robustness and generalizability of our model's diagnostic performance across different patient populations and imaging modalities. Furthermore, exploring the comparative performance of machine learning models versus radiologists or using our model as a second reader could be a promising direction for future investigations. This approach could help assess the value of machine learning-based models as an adjunct to human expertise in medical diagnostics, potentially improving both accuracy and efficiency in clinical practice.

## Data Availability

The original contributions presented in the study are included in the article/Supplementary Material, further inquiries can be directed to the corresponding author.
